# The power of a healthy lifestyle for cancer prevention: the example of colorectal cancer

**DOI:** 10.20892/j.issn.2095-3941.2022.0397

**Published:** 2022-12-05

**Authors:** Xuechen Chen, Jie Ding, Hengjing Li, Prudence R. Carr, Michael Hoffmeister, Hermann Brenner

**Affiliations:** 1 Division of Clinical Epidemiology and Aging Research, German Cancer Research Center (DKFZ), Heidelberg 69120, Germany; 2 Medical Faculty Heidelberg, Heidelberg University, Heidelberg 69120, Germany; 3 School of Public Health and Preventive Medicine, Monash University, Melbourne, VIC 3004, Australia; 4 German Cancer Consortium (DKTK), German Cancer Research Center (DKFZ), Heidelberg 69120, Germany; 5 Division of Preventive Oncology, German Cancer Research Center (DKFZ) and National Center for Tumor Diseases (NCT), Heidelberg 69120, Germany

**Keywords:** Colorectal cancer, healthy lifestyle score, polygenic risk score, family history, genetic risk equivalent

## Abstract

**Objective:**

We aimed to directly compare the estimated effects of adherence to a healthy lifestyle with those of risk predisposition according to known genetic variants affecting colorectal cancer (CRC) risk, to support effective risk communication for cancer prevention.

**Methods:**

A healthy lifestyle score (HLS) was derived from 5 lifestyle factors: smoking, alcohol consumption, diet, physical activity, and body adiposity. The association of lifestyle and polygenic risk score (PRS) (based on 140 CRC-associated risk loci) with CRC risk was assessed with multiple logistic regression and compared through the genetic risk equivalent (GRE), a novel approach providing an estimate of the effects of adherence to a healthy lifestyle in terms of percentile differences in PRS.

**Results:**

A higher HLS was associated with lower CRC risk (4,844 cases, 3,964 controls). Those adhering to all 5 healthy lifestyle factors had a 62% (95% CI 54%–68%) lower CRC risk than those adhering to ≤ 2 healthy lifestyle factors. The estimated effect of adherence to all 5 compared with ≤ 2 healthy lifestyle factors was as strong as the effect of having a 79 percentile (GRE 79, 95% CI 61–97) lower PRS. The association between a healthy lifestyle and CRC risk was independent of PRS level but was particularly pronounced among those with a family history of CRC in ≥ 1 first-degree relative (*P*-interaction = 0.0013).

**Conclusions:**

A healthy lifestyle was strongly inversely associated with CRC risk. The large GRE indicated that CRC risk determined by polygenic risk may be offset to a substantial extent by adherence to a healthy lifestyle.

## Introduction

Epidemiological studies have identified multiple lifestyle factors associated with various cancers including colorectal cancer (CRC)^1^. However, the prevalence of “risky” lifestyle factors (e.g., smoking, unhealthy diet, and obesity) remains high or has increased in many countries^2–6^. Beyond lifestyle factors, genetic predisposition is also a major determinant of CRC risk. Polygenic risk scores (PRSs) based on a steadily increasing number of single nucleotide polymorphisms identified in genome-wide association studies are increasingly used to quantify genetic predisposition^7–10^. Although PRSs may be helpful for risk stratification in secondary prevention efforts, a danger exists in that they might be misinterpreted to suggest that CRC risk is an unmodifiable feature, thus discouraging primary prevention efforts. Therefore, whether and to what extent lifestyle factors interact with genetic risk, and to what extent increased polygenic risk can be offset by a healthy lifestyle, must crucially be demonstrated. Comparisons between the effects of individual lifestyle factors and polygenic risk have recently been conducted with the genetic risk equivalent (GRE), a novel metric to enhance effective risk communication in cancer preventive efforts^11–14^.

Previous work from our group has indicated a strong association between a healthy lifestyle score, an integrative metric of lifestyle behaviors, and lower risk of CRC in a dose-dependent manner^15,16^, in agreement with previous findings^17,18^. An estimation of the extent to which increased CRC risk, as determined by polygenic risk, could be “compensated” for by adherence to healthy lifestyle behaviors could help facilitate risk communication and better inform the public regarding the benefits of adherence to a healthy lifestyle. Therefore, this study was aimed at comparing the effects of a healthy lifestyle with the effects of genetic predisposition according to known genetic variants, by using the novel concept of the GRE.

## Materials and methods

### Study design and study population

**Figure 1:**
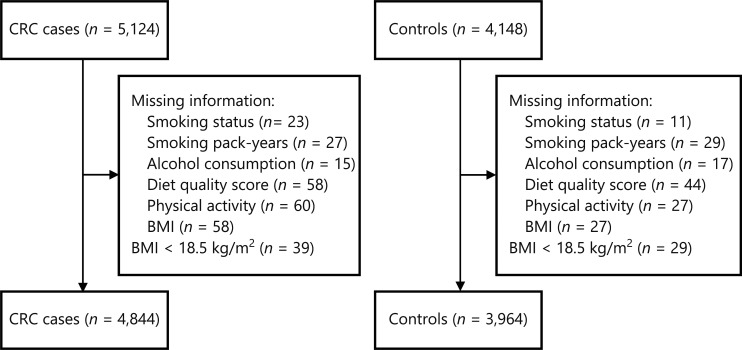
Flowchart of inclusion of study participants. BMI, body mass index; CRC, colorectal cancer.

This analysis was based on data from the DACHS [Darmkrebs: Chancen der Verhütung durch Screening (German)] study, an ongoing population-based case-control study in southwest Germany. Details of the design of the DACHS study were as reported previously^15,16,19–22^. Briefly, German-speaking patients (≥ 30 years, no upper age limit) with a first histologically confirmed diagnosis of CRC are eligible to participate. Approximately 50% of all eligible patients in the study area of approximately 2 million people are recruited from 22 hospitals offering first-line treatment to patients with CRC. Control participants are randomly selected from population registries and matched to cases by age (5-year group), gender, and county of residence. Our analyses included 4,844 cases and 3,964 controls enrolled from 2003 to 2017, for whom genetic data and complete lifestyle data were available (**Figure 1**). The DACHS study was approved by the ethics committees of the Medical Faculty of Heidelberg University (protocol code 310/2001, date of approval 06.12.2001) and the state medical boards of Baden-Wuerttemberg (protocol code M-198-02, date of approval 08.01.2003) and Rhineland-Palatinate [protocol code 837.419.02 (3637), date of approval 30.12.2002]. Written informed consent was obtained from each participant.

### Data collection

Standardized in-person interviews were scheduled during hospital stays for cases and at home for controls. Information on sociodemographic and lifestyle factors, and family and medical history was collected during interviews. Pathology records and discharge letters were obtained from medical charts for all cases. In addition, blood or buccal swab samples were collected from both cases and controls for genotyping.

Details of the lifestyle factors assessed in the DACHS study have been described in recent studies^11,13–16^. Briefly, highly detailed information on current and prior smoking behavior, including years of initiation and cessation and amounts of smoking, was obtained from each participant and used to calculate pack-years for current smokers and former smokers (defined as people who had ever smoked and had ceased for at least 2 years).

Participants were also asked about the number of alcoholic drinks [beer (0.33 L), wine (0.25 L), or liquor (0.02 L)] that they had consumed on average per week from the ages of 20 to 80 years (ascertained in 10-year intervals). On the basis of the ethanol content of each beverage type (assuming 4, 8.6, and 33 g of pure ethanol in 100 mL of beer, wine, or liquor, respectively^23^) and data from all decennial ages, we calculated the average lifetime alcohol consumption (g/d).

Dietary information was obtained with a 23-item food frequency questionnaire at baseline. Participants were asked about their average frequency of consumption over the 12 months before the date of diagnosis or interview. We calculated a diet quality score for each participant according to the updated evidence from the 2017 World Cancer Research Fund/American Institute for Cancer Research (WCRF/AICR) diet recommendations for CRC prevention^24^, as previously described by Carr et al.^15,16^. Points were assigned to 6 main food groups (red and processed meat, fish, whole grains, dairy foods, fruit, and vegetables/salad) and were then summed (**Supplementary Table S1**).

Information on the number of hours per week that participants spent performing various physical activities at the ages of 20, 30, 40, 50, 60, 70, and 80 years was obtained. Information on non-occupational physical activity (walking, cycling, or participating in sports) at the decennial age preceding the current age was used to derive the average MET min per week. We assumed 3.3, 6, and 8 MET-hours/week for each hour per week spent walking, cycling, and participating in sports, respectively^25^. We did not include occupational activity (hard exhausting work and light work) in the analysis, because most study participants were no longer occupationally active.

Participants also reported their weight at each decade from age 20 to 80 years, and their current weight and height. To avoid bias due to cancer-associated weight loss, body mass index (BMI, kg/m^2^) for this analysis was calculated on the basis of the weight approximately 10 years before the diagnosis or interview; e.g., weight at age 50 years was used for participants 55–64 years of age, weight at age 60 years was used for participants 65–74 years of age, etc.

### Derivation of the healthy lifestyle score

We calculated the healthy lifestyle score as previously proposed by Carr et al.^15,16^, including the 5 lifestyle factors of smoking, alcohol consumption, diet quality, physical activity, and BMI. Details on the derivation of the healthy lifestyle score have been published elsewhere and are summarized in **Supplementary Table S2**. Briefly, participants were assigned 1 point for the following low-risk lifestyle behaviors: non-smoking (never smoking or former smoking of < 30 pack-years^26^), alcohol consumption below the recommended level by WCRF/AICR (≤ 24 g/day for men and ≤ 12 g/day for women)^1^, a healthy diet (diet quality score in the highest 40%), being physically active (meeting the World Health Organization Global Recommendations on Physical Activity for Health: ≥ 150 min of moderate-intensity or ≥ 75 min of vigorous-intensity physical activity per week, or ≥ 500 MET min of moderate and vigorous-intensity physical activity)^27^, and having a healthy weight (BMI ≥ 18.5 to < 25 kg/m^2^). The number of points for the 5 lifestyle factors were then summed to obtain a healthy lifestyle score, which ranged from 0 (least healthy) to 5 (most healthy).

### Derivation of the polygenic risk score

DNA for genotyping was obtained from blood samples (99.1%) or from buccal swabs when blood samples were not available (0.9%). **Supplementary Table S3** presents the details on genotyping and imputation methods. The PRS in the current analysis integrates information from 140 CRC-associated risk variants identified in a recent genome-wide association study^10^ and extracted from our datasets (**Supplementary Table S4**). The unweighted score was calculated by summation of the number of risk alleles of the respective variants (0, 1, or 2 copies of the risk allele for genotyped loci; imputed dosages for imputed loci).

A weighted PRS that summed all risk alleles with weights [log odds ratio (OR) of the respective risk variants] was additionally calculated for comparisons of the associations of unweighted and weighted PRS with CRC risk. Because the results were similar (**Supplementary Table S5**), the unweighted PRS was used in all further analyses.

### Derivation of the genetic risk equivalent

GREs for individual low-risk lifestyle factors and different levels of the healthy lifestyle score were calculated as ratios of respective coefficients for healthy lifestyles and PRS percentiles from logistic regression models. The concept of GRE was developed in analogy with the well-established concept of risk and rate advancement periods^28^. Details on the calculation of GREs and 95% confidence intervals (CIs) for GREs have been published recently^11–14^. Briefly, consider an analysis based on a multivariable logistic regression:

$$\ln(\text{R})=a+b1\cdot H+b2\cdot P+\sum_{i=1}^{n}ci\cdot Fi$$

where ln(R) reflects the log odds of the disease risk, and *a*, *b*_1_, *b*_2_, and *c_i_* (*i* = 1, …, *n*) refer to the intercept and model parameters for *H* (individual healthy lifestyle factors or combined healthy lifestyles that were quantified by a healthy lifestyle score, categorized as 1 for subgroups with more healthy lifestyles and 0 for the reference group), *P* (PRS percentiles according to the distribution of PRS among controls), and *F* (other covariates). The GRE is calculated as the ratio of *b*_1_ and *b*_2_, the estimated coefficients for healthy lifestyle categories and the PRS from the regression models, and thus the properties of GRE follow from the properties of *b*_1_ and *b*_2_, which include consistency, asymptotic unbiasedness, and normality. With the delta method^29^, the asymptotic variance of GRE can be derived as:

$$\text{var}\text{(GRE)}=\frac{1}{b_{2}{}^{2}}[\text{var}(b_{1})-2.\left(\frac{b_{1}}{b_{2}}\right).\text{cov}\left(b_{1},b_{2}\right)+{\left(\frac{b_{1}}{b_{2}}\right)}^{2}.\mathrm{var}(b_{2})]$$

Because the GRE is asymptotically normal, its 95% CI can be calculated with the square root of var(GRE):

$$\text{GRE}\pm \text{1.96}\sqrt{\text{var(GRE)}}$$

As illustrated in **Supplementary Figure S1**, the assumption of a linear relationship between PRS percentiles and the log (OR) of CRC risk appears reasonable (*P* value for linear trend = 0.00066, adjusted R-squared = 0.9822), thus indicating that GREs can be interpreted in a straightforward manner. For example, a GRE of −30 for non-smoking means that the effect of abstaining from smoking would correspond to the effect of having a 30 percentile lower PRS for CRC.

### Statistical analysis

The distribution of the characteristics of cases and controls was described, and differences were compared between groups with chi-square or t tests. We also described the frequency of the healthy lifestyle factors, and measured agreement among the lifestyle factors in cases and controls by using Cohen’s kappa statistic^30^.

To assess the associations of the individual lifestyle factors (smoking, alcohol consumption, diet quality, physical activity, and BMI) with CRC risk, we used logistic regression models adjusted for the matching factors age and gender. Age was defined as age at diagnosis for cases and age at interview for controls. In further multivariable models, we additionally adjusted for education (< 9, 9–10, or > 10 years of schooling), family history of CRC (family history of CRC in a first-degree relative, yes/no), history of colonoscopy (yes/no), participation in routine health check-ups (yes/no), regular use (≥ 2 times/week for at least 1 year) of nonsteroidal anti-inflammatory drugs (NSAIDs, yes/no), and the PRS (per 10 percentiles, continuous variable). Furthermore, we included mutual adjustment for the other lifestyle factors.

Associations of the healthy lifestyle score with CRC risk was assessed in models adjusted for the same covariates described above except for mutual adjustment of the individual lifestyle factors. The healthy lifestyle score was added as a categorical variable (0–2, 3, 4, or 5 points) by using those with a score ≤ 2 as the reference group, accounting for the reasonable sample size and robust parameter estimation, or as an ordinal variable (per 1-point increase in the score; linear trend). We also evaluated the association of low, moderate, and high PRS levels (categorized according to tertiles of PRS among controls) with CRC risk, and tested for interaction with the healthy lifestyle score on CRC risk by adding a cross-product term along with the main effect terms in multivariable models. Stratified analysis of the associations between the lifestyle score and CRC by PRS level was also conducted. We performed subgroup analyses according to cancer site (colon/rectum) and clinical stage (stage I–IV), and by other potentially effect modifying factors including age (< 55 or ≥ 55 years), gender (female/male), history of colonoscopy (yes/no), use of NSAIDs (yes/no), and family history of CRC (yes/no).

All analyses were conducted in R (version 4.1.3) and SAS (version 9.4) software. All statistical tests were conducted two-sided with an alpha value of 0.05.

## Results

### Baseline characteristics of the study population by case and control status

**Table 1** presents the main characteristics of 4,844 cases and 3,964 controls. The median age was 69 years, and approximately 60% of participants were male in the case and control groups. Compared with controls, cases were less educated, were more likely to be current and former smokers (pack-years ≥30), drank more alcohol (only for male cases), were more likely to have a lower diet quality score and lower physical activity levels, and were more often overweight or obese. Healthy lifestyle scores were therefore lower for cases than for controls. More than half the cases and controls adhered to at least 3 healthy lifestyle factors, and 7.3% of cases and 14.1% of controls adhered to all 5 healthy lifestyle factors. In addition, a higher proportion of cases reported a family history of CRC, and a lower proportion of cases than controls had had a colonoscopy examination, participated in routine health check-ups, or used NSAIDs before diagnosis. Most cases had cancer in the colon (colon 60.9%; rectum 39.1%) and early-stage cancer (stage I 22.9%; stage II 30.5%; stage III 31.6%; stage IV 14.2%). The distribution of the PRS in cases and controls is shown in **Supplementary Figure S2**. CRC cases had a significantly higher PRS than controls (mean: 138.5 *vs*. 135.9, *P*-value from Kruskal-Wallis test < 0.0001), although the distributions widely overlapped.

**Table 1:** Characteristics of the study population according to case and control status

Characteristics	CRC cases, *n* (%)	Controls, *n* (%)	*P* value^8^
Total	4,844	3,964	
Age (years), median (Q1, Q3)	69 (61, 76)	69 (62, 76)	
Gender			
Female	1,897 (39.2)	1,517 (38.3)	
Male	2,947 (60.8)	2,447 (61.7)	
School education (years)^1^			<0.0001
< 9	3,172 (65.5)	2,185 (55.5)	
9–10	855 (17.7)	837 (21.1)	
> 10	808 (16.7)	936 (23.6)	
Smoking			<0.0001
Current or former (≥ 30 pack years)	1,081 (22.3)	706 (17.8)	
Never or former (< 30 pack years)	3,763 (77.7)	3,258 (82.2)	
Alcohol consumption (g/d), mean (SE)^2^			
Women	5.1 (0.2)	5.7 (0.2)	<0.0001
Men	22.4 (0.4)	19.2 (0.4)	<0.0001
Diet quality score, mean (SE)	30.5 (0.1)	32.1 (0.1)	<0.0001
Physical activity (MET-hours/week), mean (SE)^3^	40.3 (0.6)	45.8 (0.7)	<0.0001
BMI (kg/m^2^)^4^			<0.0001
Overweight or obese (≥ 25)	3,403 (70.3)	2,460 (62.1)	
Healthy weight (18.5 to < 25)	1,441 (29.7)	1,504 (37.9)	
Healthy lifestyle score			<0.0001
0	54 (1.1)	18 (0.5)	
1	345 (7.1)	174 (4.4)	
2	1,130 (23.3)	686 (17.3)	
3	1,726 (35.6)	1,347 (34.0)	
4	1,237 (25.5)	1,182 (29.8)	
5	352 (7.3)	557 (14.1)	
Family history of CRC^5^	710 (14.7)	431 (10.9)	<0.0001
History of colonoscopy	1,282 (26.5)	2,386 (60.2)	<0.0001
Participation in routine health check-ups^6^	4,123 (85.1)	3,646 (92.0)	<0.0001
Regular use of NSAIDs	1,394 (28.8)	1,509 (38.1)	<0.0001
Cancer sites			
Colon cancer	2,950 (60.9)	/	
Rectum cancer	1,894 (39.1)	/	
Cancer stages^7^			
I	1,110 (22.9)	/	
II	1,476 (30.5)	/	
III	1,529 (31.6)	/	
IV	686 (14.2)	/	

^1^Data are missing for 15 participants. ^2^Lifetime average alcohol consumption, calculated on the basis of self-recalled alcohol consumption at the ages of 20, 30, 40, 50, 60, 70, and 80 years. ^3^Non-occupational physical activity from the most recent decade preceding the date of diagnosis/interview. ^4^BMI at approximately 10 years before diagnosis/interview. ^5^Data are missing for 6 participants. ^6^Data are missing for 32 participants. ^7^Data are missing for 43 cases. ^8^*P* values are not reported for the matching factors. BMI, body mass index; CRC, colorectal cancer; MET, metabolic equivalent task; NSAID, non-steroidal anti-inflammatory drug; Q, quartile; SE, standard error.

As shown in **Supplementary Table S6**, the most prevalent healthy lifestyle factor was adherence to physical activity recommendations (cases: 84.3%; controls: 87.6%), whereas the adherence was lowest for BMI (cases: 29.7%; controls: 37.9%). With the exception of smoking and BMI, the healthy lifestyle factors tended to show slight positive agreement within participants; the highest agreement was observed between non-smoking and adherence to alcohol recommendations (kappa coefficient = 0.13 and 0.12 in cases and controls, respectively).

### Association of individual lifestyle factors with CRC risk

All low-risk lifestyle factors except adherence to physical activity recommendations (OR 0.95, 95% CI 0.83–1.09) were significantly associated with a lower risk of CRC. Multivariate adjusted ORs (95% CI) were 0.86 (0.76–0.97) for non-smoking, 0.85 (0.76–0.95) for adherence to alcohol recommendations, 0.69 (0.63–0.76) for a healthy diet quality score, and 0.67 (0.60–0.74) for a healthy BMI (**Table 2**). None of the interactions between the individual lifestyle factors and PRS on CRC risk reached statistical significance.

**Table 2:** ORs and GREs for risk of colorectal cancer according to individual lifestyle factors

Lifestyle factor	Points	Description	CRC cases, *n* (%)	Controls, *n* (%)	OR (95% CI)^1^	OR (95% CI)^2^	GRE (95% CI)
Smoking	0	Smoking: current smoker or former smoker (≥ 30 pack years)	1,079 (22.4)	704 (17.9)	Ref.	Ref.	Ref.
	1	Non-smoking: never smoker or former smoker (< 30 pack years)	3,735 (77.6)	3,237 (82.1)	0.75 (0.67, 0.83)	0.86 (0.76, 0.97)	−12.3 (−22.5, −2.2)
*P*-interaction with PRS^3^ = 0.61							
Alcohol consumption	0	Did not meet recommendations on alcoholic drinks^4^	1,266 (26.3)	911 (23.1)	Ref.	Ref.	Ref.
	1	Met recommendation on alcoholic drinks^4^	3,548 (73.7)	3,030 (76.9)	0.83 (0.75, 0.92)	0.85 (0.76, 0.95)	−13.3 (−22.6, −4.0)
*P*-interaction with PRS^3^ = 0.45							
Diet quality	0	Unhealthy diet quality: diet quality score < 34^5^	3,190 (66.3)	2,138 (54.3)	Ref.	Ref.	Ref.
	1	Healthy diet quality: diet quality score ≥ 34^5^	1,624 (33.7)	1,803 (45.7)	0.59 (0.54, 0.65)	0.69 (0.63, 0.76)	−30.4 (−39.4, −21.3)
*P*-interaction with PRS^3^ = 0.17							
Physical activity	0	Did not meet physical activity guidelines^6^	753 (15.6)	486 (12.3)	Ref.	Ref.	Ref.
	1	Met physical activity guidelines^6^	4,061 (84.4)	3,455 (87.7)	0.75 (0.66, 0.84)	0.95 (0.83, 1.09)	−4.2 (−15.6, 7.2)
*P*-interaction with PRS^3^ = 0.35							
BMI	0	Overweight or obese (BMI ≥ 25 kg/m^2^)	3,384 (70.3)	2,447 (62.1)	Ref.	Ref.	Ref.
	1	Healthy weight (18.5 < BMI < 25 kg/m^2^)	1,430 (29.7)	1,494 (37.9)	0.67 (0.61, 0.74)	0.67 (0.60, 0.74)	−32.8 (−42.2, −23.3)
*P*-interaction with PRS^3^ = 0.36							

^1^Adjusted for matching factors age and gender. ^2^Additionally adjusted for school education, family history of CRC, history of colonoscopy, participation in routine health check-ups, use of nonsteroidal anti-inflammatory drugs, PRS (per 10 percentiles, continuous), and mutual adjustment for the other lifestyle factors. ^3^Interactions were tested by inclusion of a cross-product term of the PRS (continuous variable) and the individual lifestyle factors (categorical variable) along with the main effect terms in multivariable models. ^4^World Cancer Research Fund/American Institute for Cancer Research (WCRF/AICR) (2007) Recommendation on alcoholic drinks: ≤ 24 g/day for men and ≤ 12 g/day for women. ^5^Diet quality score in the highest 40%. ^6^The WHO Global Recommendations on Physical Activity for Health (2010) recommend that adults engage in at least 150 min of moderate-intensity or 75 min of vigorous-intensity aerobic physical activity throughout the week, or an equivalent combination of moderate and vigorous intensity physical activity (at least ∼500 MET min). BMI, body mass index; CI, confidence intervals; CRC, colorectal cancer; GRE, genetic risk equivalent; OR, odds ratio; PRS, polygenic risk score; Ref., reference.

### Association of the healthy lifestyle score with CRC risk

In combined analyses, the healthy lifestyle score was inversely associated with CRC risk independently of PRS level (**Table 3**). Participants with a healthy lifestyle score of 3, 4, or 5 points had a 22% (95% CI 12% to 31%), 37% (95% CI 28% to 45%), and 62% (95% CI 54% to 68%) lower risk of CRC than those with a healthy lifestyle score ≤ 2 points. These associations were similar in each PRS tertile (**Supplementary Table S7**) and in subgroups stratified by cancer site (**Table 4**), age, gender, history of colonoscopy, and use of NSAIDs, but varied by family history of CRC (**Supplementary Tables S8 and S9**). The highest healthy lifestyle score was associated with an 80% lower risk of CRC among participants with a family history of CRC (OR 0.20, 95% CI 0.11–0.33), thus indicating a much stronger risk reduction than that among those without a family history of CRC (OR 0.42, 95% CI 0.35–0.51) (**Supplementary Table S9**). We observed a stronger risk reduction of stage IV CRC with adherence to all 5 healthy lifestyle factors compared with stage I–III CRC (*P* value for heterogeneity = 0.0018, **Table 4**).

**Table 3:** Individual associations of the polygenic risk score and the healthy lifestyle score with colorectal cancer risk

Variables	CRC cases, *n* (%)	Controls, *n* (%)	OR (95%CI)^1^	OR (95% CI)^2^	GRE (95% CI)
PRS^3^					
Low	1,026 (21.3)	1,317 (33.4)	Ref.	Ref.	
Moderate	1,544 (32.1)	1,313 (33.3)	1.51 (1.35, 1.68)	1.54 (1.37, 1.74)	
High	2,244 (46.6)	1,311 (33.3)	2.20 (1.97, 2.44)	2.21 (1.97, 2.48)	
PRS (per 10 percentile increase)			1.13 (1.11, 1.15)	1.13 (1.11, 1.15)	
Healthy lifestyle score					
0–2	1,519 (31.6)	876 (22.2)	Ref.	Ref.	
3	1,715 (35.6)	1,337 (33.9)	0.72 (0.65, 0.81)	0.78 (0.69, 0.88)	−20.3 (−30.6, −10.0)
4	1,233 (25.6)	1,175 (29.8)	0.57 (0.51, 0.65)	0.63 (0.55, 0.72)	−37.8 (−49.7, −25.9)
5	347 (7.2)	553 (14.0)	0.33 (0.28, 0.39)	0.38 (0.32, 0.46)	−79.2 (−97.3, −61.1)
Per 1-point increase			0.74 (0.71, 0.78)	0.78 (0.74, 0.81)	−20.3 (−25.0, −15.7)
*P* value for interaction between PRS and healthy lifestyle score^4^ = 0.88/0.39					

^1^Adjusted for age and gender. ^2^Additionally adjusted for school education, family history of CRC, history of colonoscopy, participation in routine health check-ups, use of nonsteroidal anti-inflammatory drugs, healthy lifestyle score (categorical variable, for the analysis of PRS), and PRS (continuous variable with per 10 percentile increase, for the analysis of healthy lifestyle score). ^3^PRS was categorized into low, moderate, and high levels according to tertiles of PRS among controls. ^4^Interactions were tested by inclusion of a cross-product of the PRS (categorical variable/continuous variable) and the healthy lifestyle score (categorical variable) along with the main effect terms in multivariable models. CI, confidence intervals; CRC, colorectal cancer; OR, odds ratio; PRS, polygenic risk score; Ref., reference.

**Table 4:** ORs and GREs for colorectal cancer risk according to the healthy lifestyle score in subgroups by cancer site and stage

CRC	Healthy lifestyle score	Cases, *n* (%)	Controls, *n* (%)	OR (95% CI)^1^	GRE (95% CI)
Colon cancer	0–2	885 (30.2)	876 (22.2)	Ref.	Ref.
	3	1,060 (36.2)	1,337 (33.9)	0.79 (0.69, 0.91)	−19.3 (−30.7, −7.8)
	4	770 (26.3)	1,175 (29.8)	0.63 (0.55, 0.73)	−37.8 (−51.1, −24.6)
	5	217 (7.4)	553 (14.0)	0.39 (0.32, 0.47)	−77.0 (−97.3, −56.8)
	Per 1-point increase			0.78 (0.74, 0.82)	−20.3 (−25.5, −15.1)
Rectal cancer	0–2	634 (33.7)	876 (22.2)	Ref.	Ref.
	3	655 (34.8)	1,337 (33.9)	0.75 (0.64, 0.88)	−22.0 (−34.7, −9.2)
	4	463 (24.6)	1,175 (29.8)	0.61 (0.51, 0.73)	−37.7 (−52.5, −23.0)
	5	130 (6.9)	553 (14.0)	0.37 (0.29, 0.48)	−75.9 (−98.7, −53.0)
	Per 1-point increase			0.77 (0.73, 0.82)	−19.9 (−25.7, −14.2)
*P* value for heterogeneity between strata (3/4/5 points) = 0.41/0.57/0.83					
CRC (stage I)	0–2	309 (28.0)	876 (22.2)	Ref.	Ref.
	3	401 (36.3)	1,337 (33.9)	0.87 (0.72, 1.04)	−11.4 (−26.5, 3.7)
	4	303 (27.4)	1,175 (29.8)	0.76 (0.62, 0.92)	−22.5 (−39.3, −5.6)
	5	92 (8.3)	553 (14.0)	0.49 (0.37, 0.64)	−58.4 (−84.0, −32.7)
	Per 1-point increase			0.84 (0.79, 0.90)	−14.3 (−20.7, −7.9)
CRC (stage II)	0–2	501 (34.1)	876 (22.2)	Ref.	Ref.
	3	548 (37.3)	1,337 (33.9)	0.74 (0.62, 0.87)	−24.6 (−39.0, −10.3)
	4	324 (22.1)	1,175 (29.8)	0.47 (0.39, 0.57)	−61.8 (−81.2, −42.4)
	5	96 (6.5)	553 (14.0)	0.32 (0.24, 0.42)	−93.2 (−121.7, −64.8)
	Per 1-point increase			0.71 (0.67, 0.76)	−28.0 (−35.6, −20.4)
CRC (stage III)	0–2	464 (30.6)	876 (22.2)	Ref.	Ref.
	3	505 (33.3)	1,337 (33.9)	0.74 (0.63, 0.88)	−24.6 (−39.1, −10.1)
	4	423 (27.9)	1,175 (29.8)	0.67 (0.56, 0.80)	−32.8 (−48.7, −16.9)
	5	124 (8.2)	553 (14.0)	0.43 (0.33, 0.55)	−69.1 (−93.3, −44.8)
	Per 1-point increase			0.81 (0.76, 0.86)	−17.2 (−23.4, −11.1)
CRC (stage IV)	0–2	234 (34.4)	876 (22.2)	Ref.	Ref.
	3	244 (35.8)	1,337 (33.9)	0.77 (0.62, 0.96)	−21.4 (−40.1, −2.6)
	4	170 (25.0)	1,175 (29.8)	0.60 (0.47, 0.76)	−41.8 (−64.6, −19.0)
	5	33 (4.8)	553 (14.0)	0.25 (0.16, 0.37)	−113.4 (−157.8, −69.0)
	Per 1-point increase			0.72 (0.66, 0.79)	−26.9 (−36.8, −17.0)
*P* value for heterogeneity between strata (3/4/5 points) = 0.23/0.00015/0.0018					

^1^Variables in the model included age, gender, school education, family history of CRC, history of colonoscopy, participation in routine health check-ups, use of nonsteroidal anti-inflammatory drugs, healthy lifestyle score, and PRS (per 10 percentiles, continuous). CI, confidence intervals; CRC, colorectal cancer; GRE, genetic risk equivalent; OR, odds ratio; PRS, polygenic risk score; Ref., reference.

### Genetic risk equivalents for different levels of the healthy lifestyle score

Each point increase in the healthy lifestyle score was equivalent to a decrease in CRC risk corresponding to a 20 percentile lower ranking in the PRS (GRE −20, 95% CI −25 to −16) (**Table 3**). The estimated combined effect of adherence to all 5 healthy lifestyle factors was equivalent to that of having a 79 percentile (GRE −79, 95% CI −97 to −61) lower PRS. Similar GREs were estimated for colon and rectal cancer (**Table 4**), and in subgroups defined by age, gender, history of colonoscopy, or use of NSAIDs (**Supplementary Tables S8 and S9**). Again, the most pronounced GREs were estimated for those with a family history of CRC, among whom an increase in healthy lifestyle score by 1 point was equivalent to a 34 percentile (GRE −34, 95% CI −47 to −20) lower ranking in the PRS distribution (**Supplementary Table S9**).

## Discussion

In this large population-based case-control study, a healthy lifestyle score incorporating information from known lifestyle factors was associated with a lower risk of CRC in a dose-dependent manner, regardless of polygenic risk of CRC. Those adhering to all 5 healthy lifestyle factors had a 62% (95% CI 54%–68%) lower risk of CRC than those adhering to ≤ 2 healthy lifestyle factors. The effect of adhering to all 5 healthy lifestyle factors compared with ≤ 2 healthy lifestyle factors was estimated to be as strong as the effect of having a 79 (95% CI 61–97) percentile lower PRS. Intriguingly, the estimated effects of a healthy lifestyle were more evident among participants who reported a family history of CRC. The large GREs for individuals with a healthy lifestyle underscores the benefits of adherence to lifestyle recommendations in CRC prevention.

Several previous analyses^15–18^ have explored the interaction of lifestyle scores and PRS on CRC risk, all of which observed a similar pattern of effects of combined lifestyle factors on CRC at different PRS levels. In our analysis, we used a combination of available international recommendations as well as study specific cutoffs for the determination of the healthy lifestyle score. Moreover, we used the same definition of the healthy lifestyle score as that in previous work by Carr et al.^15,16^, which was based on a smaller data set from the DACHS study available at that time. Our study corroborates and extends the results of these previous analyses, which also had included comprehensive sensitivity analyses, by adding comparative analyses of the effects of PRS and individual and combined healthy lifestyle factors on CRC risk in a larger sample of cases and controls. Cho et al.^17^ have calculated a combined lifestyle risk score based on 5 modifiable factors (obesity, physical activity, smoking, alcohol consumption, and dietary inflammatory index) and have observed that a lifestyle risk score in the highest tertile was associated with an approximately 5.8-fold greater risk of CRC than the score in the lowest tertile. In a study by Choi et al.^18^, healthy lifestyle scores were constructed by using 8 lifestyle factors, primarily according to the American Cancer Society guidelines. A score ≥ 4 points was associated with a 29% (95% CI 19% to 37%) lower risk of CRC than a score ≤ 1 point. A recent study based on 2 large international consortia (including DACHS data) from 1992 to 2005 has developed an “E-score” involving 19 lifestyle and environmental risk factors, and has observed a greater CRC risk with higher E-scores—an effect also independent of PRS level^31^. Although the definitions of the lifestyle scores varied, and the number of risk variants involved in the PRS construction also differed among studies (the numbers of variants were smaller than in our study and varied between 13 and 95 in previous studies), all these findings underscore the importance of adherence to lifestyle recommendations regardless of polygenic risk of CRC.

An intriguing finding in our analysis was a notable variation in lifestyle-CRC associations according to family history status. Although family history, like PRS, reflects genetic predisposition to some extent, it may also reflect shared environmental factors. In our study, family history of CRC was associated with less healthy lifestyle factors; this finding may partly reflect the clustering of risky lifestyle behaviors within families. Another aspect requiring careful consideration is that family history may also be associated with rare variants with high penetrance (e.g., mutations of APC tumor suppressor genes and DNA mismatch repair genes), whereas PRSs are built on the basis of common risk variants with low penetrance^32,33^. Therefore, family history and PRS may partly represent 2 different and complementary sources of genetic risk. Interestingly, interactions between lifestyle factors and rare genetic variants with respect to CRC risk have been reported in previous studies^34,35^; therefore, such interactions might also have contributed to the interactions between family history and lifestyle factors observed in our study. Further large-scale studies are necessary to validate these findings, and to further decipher the genetic and environmental components of family history and clarify their interactions with healthy lifestyles in colorectal carcinogenesis.

However, no studies to date have directly compared the magnitude of CRC risk associated with a combined healthy lifestyle score to the magnitude of CRC risk increased by known genetic variants. Communicating genetic risk in ways that could maximize understanding and promote public health is essential but challenging for diseases resulting from the complex interplay between genetic and environmental factors, particularly as genetic information is rapidly emerging with advances in genomic technologies. The GRE might serve as a useful supplementary metric to the traditional approaches commonly used to quantify the association of exposure with the risk of a specific outcome, such as odds ratios, whose meaning may be difficult to explain to laypeople and thus may hinder effective risk communication. Communicating the effects of modifiable risk factors of CRC in terms of GREs might help individuals feel less powerless against their genetic predisposition to CRC and empower them to adhere to healthy lifestyle recommendations.

A major strength of our study is its use of a large sample size and detailed information on the participants’ lifestyles as well as a comprehensive set of other CRC-associated factors, which enabled thorough confounder adjustment and detailed subgroup analysis. Our study adds important information to the limited evidence on the interaction between individual and combined healthy lifestyle factors and polygenic risk of CRC. Furthermore, this is the first study deriving GREs for different levels of healthy lifestyles, which might help promote effective risk communication in cancer prevention.

Despite these strengths, several limitations of our study also require careful consideration, particularly those resulting from the case-control design of this study. First, we cannot rule out the possibility of information bias, because most of the data, including information on lifestyle factors, were retrospectively gathered. Imperfect recall or imprecise reporting might have attenuated the associations. Second, we cannot rule out the possibility of selection bias: those who participated in our study might potentially have tended to be more health-conscious than those who did not. In particular, overrepresentation of healthier controls included in the analyses might have led to an overestimation of lifestyle-CRC associations. However, adjustment for several covariates associated with health consciousness, such as education, history of colonoscopy examination, and history of routine health check-ups, in the regression models should have limited potential bias from this source. Third, despite comprehensive covariate adjustment, residual confounding by omitted or imperfectly measured confounders cannot be ruled out. Fourth, despite the overall large sample size, the sample size in certain subgroups, such as the younger population and the subgroup with a family history of CRC, was relatively small, thus resulting in wide confidence intervals for risk estimates and GREs in these groups. Finally, the results in our study have not been validated in different populations and were based on a population of almost exclusively European ancestry. Further studies are warranted to validate our results in larger populations, and in populations with other or more diverse ethnicities.

## Conclusions

In conclusion, we observed a substantial decrease in risk with adherence to combined healthy lifestyle factors; this effect was independent of the polygenic risk of CRC but was more apparent among those with a family history of CRC. A comparably strong risk reduction in relative terms at all levels of PRS implied a particularly strong absolute risk reduction associated with a high healthy lifestyle score for individuals with a high PRS^16^. The large GRE estimates indicated that a high polygenic risk of CRC can be offset to a substantial extent by a healthy lifestyle and can be greatly “compensated” for by adherence to healthy lifestyle recommendations. These findings might help inform targeted CRC prevention efforts and motivate adherence to healthy lifestyle recommendations. Future studies and further validation are warranted to replicate and corroborate our findings and to provide more precise GREs, particularly for the high-risk group with a family history of CRC.
