# Supplementary material for: The power of a healthy lifestyle for cancer prevention: the example of colorectal cancer
Source: Cancer Biol Med. 2022 Dec 5;19(11):1586–97. doi: 10.20892/j.issn.2095-3941.2022.0397 (PMC9724224; doi:10.20892/j.issn.2095-3941.2022.0397)
Supplement: Supplementary file 1 [file cbm-19-1586-s001.pdf]

Supplementary materials

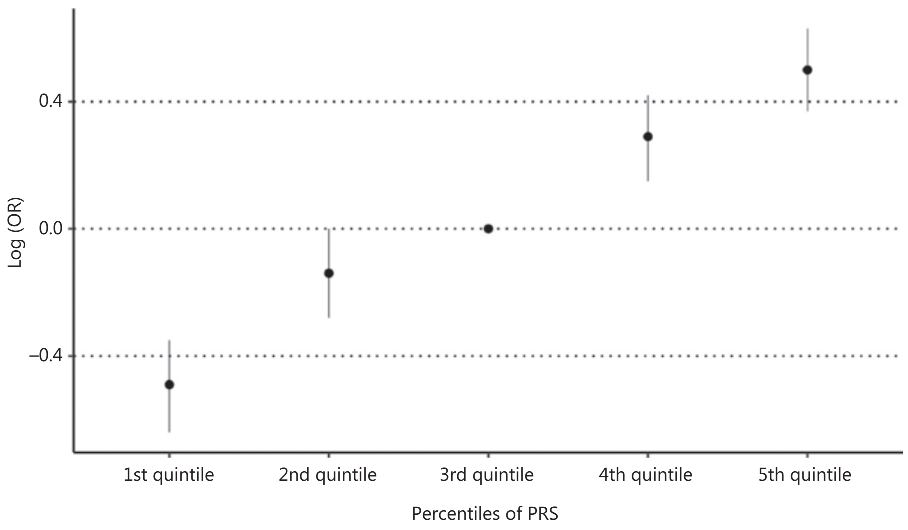

Figure S1 Log odds ratio of colorectal cancer risk according to polygenic risk score level. OR, odds ratio; PRS, polygenic risk score.

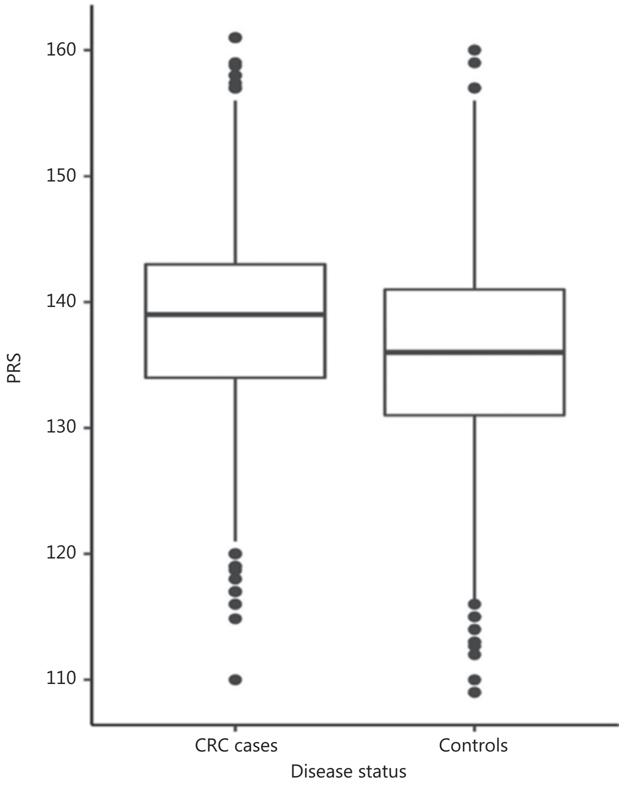

Figure S2 Boxplot of polygenic risk scores among colorectal cancer cases and controls. CRC, colorectal cancer; PRS, polygenic risk score.

Table S1 Composition and points for the diet quality score, on the basis of responses from the DACHS study food frequency questionnaire

Food groups	Food frequency questionnaire responses						Total maximum
	Multiple times per day	Once per day	Multiple times per week	Once per week	Less than once per week	Never	
Red and processed meat[1]	0	1	2	3	4	5	5
Fish	5	4	3	2	1	0	5
Whole grains[2]	10	8	6	4	2	0	10
Dairy foods[3]	10	8	6	4	2	0	10
Fruit	10	8	6	4	2	0	10
Vegetables/salad	10	8	6	4	2	0	10
							50

Note: This table was developed by Carr et al. (PMID: 30201362; PMID: 32179093). [1]Red and processed meat: fresh pork, beef, etc., sausages made from beef or pork, luncheon meats (e.g., salami/sliced sausage), or ham. [2]Whole grains and whole grain products: e.g., whole grain bread, muesli, or other whole grain products. [3]Dairy foods: cheese or quark, yogurt, or milk.

Table S2 Description of the individual lifestyle factors comprising the healthy lifestyle score

Lifestyle factor	Points	Description
Smoking	0	Smoking: current smoker or former smoker (≥ 30 pack years)
	1	Non-smoking: never smoker or former smoker (< 30 pack years)
Alcohol consumption	0	Did not meet recommendations on alcoholic drinks[1]
	1	Met recommendation on alcoholic drinks[1]
Diet quality	0	Unhealthy diet quality: diet score[2] < diet score in the highest 40%
	1	Healthy diet quality: diet score[2] ≥ diet score in the highest 40%
Physical activity	0	Did not meet physical activity guidelines[3]
	1	Met physical activity guidelines[3]
Body mass index	0	Overweight or obese (BMI ≥ 25 kg/m^2)
	1	Healthy weight (18.5 < BMI < 25 kg/m^2)

Note: This table was developed by Carr et al. (PMID: 30201362; PMID: 32179093). [1]World Cancer Research Fund/American Institute for Cancer Research (WCRF/AICR) (2007) Recommendation on alcoholic drinks: ≤ 24 g/day for men and ≤ 12 g/day for women. [2]Calculated on the basis of data from the Food Frequency Questionnaires according to the updated evidence from the 2017 WCRF/AICR diet recommendations for CRC prevention. [3]The World Health Organization Global Recommendations on Physical Activity for Health (2010) recommend that adults engage in at least 150 min of moderate-intensity or 75 min of vigorous-intensity aerobic physical activity throughout the week, or an equivalent combination of moderate and vigorous intensity physical activity (at least ~500 MET minutes). BMI, body mass index.

Table S3 Information on genotyping and imputation

Genotyping platform	CRC cases (*n*)	Controls (*n*)	Recruitment Period	Imputation
Illumina HumanCytoSNP	1,588	1640	2003–2008	
Illumina HumanOmniExpress	649	473	2007–2010	
Illumina HumanOmniExpress	1,128	596	2010–2015	Haplotype Reference Consortium (version r1.1.2016)
Illumina Infinium OncoArray	858	626	2003–2016	
Illumina Global Screening Array	621	629	2016–2017	

Note: We excluded triallelic SNPs, genotyped SNPs that had a low call rate (< 98%), lack of Hardy-Weinberg equilibrium in control individuals ($P < 1\times10^{-4}$), or low minor allele frequency (< 0.1%), and those not assigned an rs number. More details can be found in previous studies by Peters et al.[1] and Schumacher et al.[2]. [1]Peters U, Jiao S, Schumacher FR, et al. Identification of genetic susceptibility loci for colorectal tumors in a genome-wide meta-analysis. Gastroenterology 2013; 144:799–807. [2]Schumacher FR, Schmit SL, Jiao S, et al. Genome-wide association study of colorectal cancer identifies 6 new susceptibility loci. Nat Commun 2015; 6:7138.

Table S4 Overview of colorectal cancer related single-nucleotide polymorphisms identified in genome-wide association studies and considered in this analysis

SNP	Locus	Position	Risk allele	Beta
rs4360494	1p34.3	38455891	G	0.0379
rs12144319	1p32.3	55246035	C	0.0661
rs72647484	1p36.12	22587728	T	0.0504
rs7542665	1p31.3	62673037	C	0.0334
rs6678517	1q25.3	183002639	A	0.073
rs17011141	1q41	222112634	G	0.0877
rs448513	2q24.2	159964552	C	0.0054
rs11884596	2q33.1	199612407	C	0.0342
rs983402	2q33.1	199781586	T	0.0622
rs7606562	2p16.3	48686695	T	0.0414
rs11692435	2q11.2	98275354	G	0.0492
rs3731861	2q35	219191256	T	0.0613
rs10049390	3q22.2	133701119	A	0.0455
rs13086367	3q13.2	112903888	A	0.0463
rs72942485	3q13.2	112999560	G	0.0545
rs9831861	3p21.1	53088285	G	0.0294
rs35470271	3p22.1	40915239	G	0.0994
rs12635946	3q13.2	112916918	C	0.0334
rs113569514	3q22.2	133748789	T	0.0414
rs9876206	3q26.2	169517436	C	0.0453
rs6781752	3p14.1	66365163	A	0.0597
rs11727676	4q31.21	145659064	C	0.0093
rs1391441	4q24	106128760	A	0.0148

Table S4 Continued

SNP	Locus	Position	Risk allele	Beta
rs13149359	4q22.2	94938618	A	0.052
rs7708610	5p13.1	40102443	A	0.0384
rs78368589	5p15.33	1240204	T	0.0786
rs145364999	5q21.1	98206082	T	0.3496
rs2735940	5p15.33	1296486	G	0.0865
rs12514517	5p13.1	40280076	A	0.1013
rs755229494	5q22.2	112097351	G	0.6286
rs12659017	5q23.2	125988175	G	0.0374
rs4976270	5q31.1	134467220	C	0.0693
rs13204733	6p12.1	55566108	G	0.0643
rs116685461	6p21.33	31315512	G	0.0655
rs9271695	6p21.32	32593080	G	0.0889
rs2516420	6p21.33	31449620	C	0.1091
rs116353863	6p21.33	31010185	C	0.1202
rs16878812	6p21.31	35569562	A	0.0778
rs9470361	6p21.2	36623379	A	0.054
rs62404966	6p12.1	55712124	C	0.0724
rs3131043	6p21.33	30758466	G	0.0294
rs2070699	6p24.1	12292772	T	0.0294
rs1476570	6p22.1	29809860	A	0.0492
rs3830041	6p21.32	32191339	T	0.0645
rs6928864[1]	6q21	105966894	C	0.0531
rs62396735	6p21.1	41702582	C	0.033
rs12672022	7p13	45136423	T	0.0067

Table S4 Continued

SNP	Locus	Position	Risk allele	Beta
rs80077929	7p12.3	46094089	T	0.0093
rs10951878	7p12.3	46926695	C	0.0531
rs3801081	7p12.3	47511161	G	0.0253
rs7013278	8q24.21	128414892	T	0.0091
rs4313119	8q24.21	128571855	G	0.0518
rs16892766	8q23.3	117630683	C	0.2099
rs6469654	8q23.3	117632965	G	0.0677
rs117079142	8q24.11	117790914	A	0.1139
rs6983267	8q24.21	128413305	G	0.1052
rs34405347	9q22.33	101679752	T	0.0089
rs1537372	9p21.3	22103183	G	0.012
rs10980628	9q31.3	113671403	C	0.0511
rs12217641	10p14	8663875	C	0.0069
rs10786560	10q24.2	101315166	G	0.0082
rs1250567	10q22.3	81046265	C	0.047
rs11255841	10p14	8739580	T	0.1064
rs10821907	10q11.23	52648454	C	0.073
rs704017	10q22.3	80819132	G	0.0765
rs11190164	10q24.2	101351704	G	0.0889
rs12246635	10q25.2	114288619	C	0.0975
rs11196170	10q25.2	114722621	A	0.0527
rs7946853	11q13.4	74409077	C	0.0119
rs55864876	11q22.1	100717136	G	0.015
rs2186607	11q22.1	101656397	T	0.0483
rs61389091	11q13.4	74427921	C	0.1934
rs4450168	11p15.4	10286755	C	0.0413
rs174533	11q12.2	61549025	G	0.0636
rs7121958	11q13.4	74280012	G	0.078
rs3087967	11q23.1	111156836	T	0.1122
rs4759277	12q13.3	57533690	A	0.0285
rs1427760	12q24.21	115100714	C	0.0424
rs3217874	12p13.32	4400808	T	0.0453
rs10849433	12p13.31	6406904	C	0.0468
rs11610543	12q12	43134191	G	0.0474
rs35808169	12p13.32	4368607	C	0.089

Table S4 Continued

SNP	Locus	Position	Risk allele	Beta
rs3217810	12p13.32	4388271	T	0.1181
rs2250430	12p13.31	6421174	T	0.0597
rs77969132	12p11.21	31594813	T	0.1583
rs12372718	12q13.12	51171090	G	0.0896
rs597808	12q24.12	111973358	G	0.0737
rs7300312	12q24.21	115890922	C	0.066
rs2710310	12p13.2	12035649	C	0.0145
rs78341008	13q22.1	73791554	C	0.0109
rs8000189	13q34	111075881	T	0.0473
rs45597035	13q22.1	73649152	A	0.0495
rs1924816	13q22.1	73997961	A	0.0506
rs7333607	13q13.3	37462010	G	0.0758
rs1330889	13q22.3	78609615	C	0.0453
rs377429877	13q13.2	34092164	C	0.0468
rs1951864	14q22.2	54369299	A	0.0059
rs17094983	14q23.1	59189361	G	0.0062
rs8020436	14q23.1	59208437	A	0.0294
rs35107139	14q22.2	54419106	C	0.0912
rs4901473	14q22.2	54445157	G	0.0465
rs745213	15q23	68060389	G	0.0072
rs12594720	15q22.31	67007018	C	0.0246
rs56324967	15q22.33	67402824	C	0.0689
rs17816465	15q13.3	33156386	A	0.069
rs12708491	15q13.3	32992836	G	0.0464
rs2293581	15q13.3	33010736	A	0.1248
rs7495132	15q26.1	91172901	T	0.0453
rs9930005	16q23.2	80043258	C	0.0061
rs12447408	16q24.1	86252544	A	0.0079
rs9924886	16q22.1	68743939	A	0.055
rs12149163	16q24.1	86339315	T	0.0487
rs62042090	16q24.1	86703949	T	0.0481
rs983318	17q24.3	70413253	A	0.0397
rs73975586	17p13.3	814243	A	0.0497
rs1078643	17p12	10707241	A	0.0747
rs75954926	17q25.3	81061048	G	0.0882

Table S4 Continued

SNP	Locus	Position	Risk allele	Beta
rs373585858	17q25.3	80394556	A	0.1103
rs4968127	17p13.3	809643	G	0.0514
rs11874392	18q21.1	46453156	A	0.1606
rs73068325	19q13.43	59079096	T	0.0066
rs34797592	19p13.11	16417198	T	0.0824
rs28840750	19q13.11	33519927	T	0.1939
rs1963413	19q13.2	41871573	A	0.0441
rs12979278	19q13.33	49218602	T	0.0293
rs2738783	20q13.33	62308612	T	0.006
rs6067417	20q13.13	48983697	C	0.0331
rs6031311	20q13.12	42666475	T	0.0362
rs6091189	20q13.13	49256285	T	0.0549
rs994308	20p12.3	6603622	C	0.0626
rs28488	20p12.3	6762221	T	0.0714
rs556532366	20p12.3	8568071	T	0.0715
rs189583	20p12.3	6376457	G	0.0795
rs4813802	20p12.3	6699595	G	0.0819
rs11087784	20p12.3	7740976	G	0.0874
rs6066825	20q13.13	47340117	A	0.0719
rs6063514	20q13.13	49055318	C	0.0547
rs13831	20q13.32	57475191	G	0.0334
rs1741640	20q13.33	60932414	C	0.1146
rs6058093	20q11.22	33213196	C	0.045

[1]For building the PRS, the missing reference SNP was replaced by rs6904092 (linkage disequilibrium, D′ = 1 and r^2 = 1). A, adenine; C, cytosine; G, guanine; OR, odds ratio; T, thymine; SNP, single-nucleotide polymorphism.

Table S5 Association of unweighted and weighted polygenic risk score with colorectal cancer risk

PRS	PRS decile	CRC cases, *n* (%)	Controls, *n* (%)	OR (95% CI)[1]	OR (95% CI)[2]
Unweighted PRS	D1	215 (4.5)	395 (10.0)	Ref.	Ref.
	D2	326 (6.8)	399 (10.1)	1.44 (1.13, 1.83)	1.44 (1.14, 1.84)
	D3	334 (6.9)	390 (9.9)	1.63 (1.28, 2.07)	1.64 (1.29, 2.08)
	D4	435 (9.0)	399 (10.1)	2.01 (1.60, 2.54)	2.02 (1.60, 2.55)
	D5	372 (7.7)	395 (10.0)	1.92 (1.52, 2.44)	1.93 (1.52, 2.44)
	D6	502 (10.4)	387 (9.8)	2.31 (1.84, 2.91)	2.33 (1.85, 2.94)
	D7	604 (12.5)	395 (10.0)	2.79 (2.23, 3.51)	2.82 (2.25, 3.55)
	D8	571 (11.9)	395 (10.0)	2.74 (2.18, 3.44)	2.76 (2.20, 3.47)
	D9	617 (12.8)	392 (9.9)	2.87 (2.29, 3.60)	2.87 (2.29, 3.60)
	D10	838 (17.4)	394 (10.0)	3.91 (3.14, 4.89)	3.93 (3.15, 4.91)
Weighted PRS	D1	228 (4.7)	395 (10.0)	Ref.	Ref.
	D2	291 (6.0)	394 (10.0)	1.32 (1.03, 1.68)	1.31 (1.03, 1.67)
	D3	327 (6.8)	394 (10.0)	1.52 (1.20, 1.93)	1.52 (1.20, 1.93)
	D4	397 (8.2)	394 (10.0)	1.85 (1.47, 2.34)	1.85 (1.47, 2.33)
	D5	433 (9.0)	394 (10.0)	2.07 (1.64, 2.61)	2.06 (1.63, 2.59)
	D6	492 (10.2)	394 (10.0)	2.26 (1.80, 2.84)	2.26 (1.80, 2.84)
	D7	530 (11.0)	394 (10.0)	2.50 (1.99, 3.14)	2.52 (2.01, 3.16)
	D8	578 (12.0)	394 (10.0)	2.64 (2.11, 3.31)	2.64 (2.11, 3.31)
	D9	681 (14.1)	394 (10.0)	3.17 (2.54, 3.96)	3.19 (2.55, 3.98)
	D10	857 (17.8)	394 (10.0)	3.89 (3.13, 4.85)	3.89 (3.12, 4.84)

[1]Adjusted for smoking, alcohol intake, diet quality, physical activity, body mass index, age, gender, education, family history of CRC, history of colonoscopy, participation in routine health check-ups, and use of nonsteroidal anti-inflammatory drugs. [2]Adjusted for healthy lifestyle score, age, gender, education, family history of CRC, history of colonoscopy, participation in routine health check-ups, and use of nonsteroidal anti-inflammatory drugs. CI, confidence interval; CRC, colorectal cancer; D, decile of PRS among controls; OR, odds ratio; PRS, polygenic risk score; Ref., reference.

Table S6 Frequency of the healthy lifestyle factors and agreement among the various healthy lifestyle factors in cases and controls

Healthy lifestyle factor	CRC cases (*n* = 4,844)						Controls (*n* = 3,964)					
	n (%)	Kappa coefficient					*n* (%)	Kappa coefficient				
		SMK	ALC	DQ	PA	BMI		SMK	ALC	DQ	PA	BMI
SMK	3,763 (77.7)	1.00	0.13	0.085	0.035	−0.027	3,258 (82.2)	1.00	0.12	0.092	0.052	−0.018
ALC	3,571 (73.7)		1.00	0.050	0.010	0.034	3,051 (77.0)		1.00	0.057	−0.012	0.044
DQ	1,632 (33.7)			1.00	0.044	0.079	1,813 (45.7)			1.00	0.059	0.097
PA	4,084 (84.3)				1.00	0.040	3,474 (87.6)				1.00	0.033
BMI	1,441 (29.7)					1.00	1,504 (37.9)					1.00

Note: All healthy lifestyle factors were categorized into 2 subgroups according to pertinent health guidelines. ALC, alcohol intake; BMI, body mass index; CRC, colorectal cancer; DQ, diet quality; PA, physical activity; SMK, smoking.

Table S7 Associations of the healthy lifestyle score with colorectal cancer risk by polygenic risk score level

PRS[1]	Healthy lifestyle score	CRC cases, *n* (%)	Controls, *n* (%)	OR (95% CI)[2]
Low	0–2	313 (30.5)	264 (20.0)	Ref.
	3	374 (36.5)	457 (34.7)	0.70 (0.55, 0.88)
	4	273 (26.6)	406 (30.8)	0.58 (0.45, 0.74)
	5	66 (6.4)	190 (14.4)	0.30 (0.21, 0.43)
	Per 1-point increase			0.73 (0.67, 0.80)
Moderate	0–2	479 (31.0)	301 (22.9)	Ref.
	3	564 (36.5)	455 (34.7)	0.82 (0.66, 1.00)
	4	395 (25.6)	380 (28.9)	0.67 (0.54, 0.84)
	5	106 (6.9)	177 (13.5)	0.43 (0.32, 0.59)
	Per 1-point increase			0.80 (0.74, 0.87)
High	0–2	727 (32.4)	311 (23.7)	Ref.
	3	777 (34.6)	425 (32.4)	0.79 (0.65, 0.96)
	4	565 (25.2)	389 (29.7)	0.62 (0.50, 0.76)
	5	175 (7.8)	186 (14.2)	0.39 (0.30, 0.52)
	Per 1-point increase			0.78 (0.73, 0.84)

[1]PRS was categorized into low, moderate, and high levels according to tertiles of PRS among controls. [2]Adjusted for age, gender, school education, family history of CRC, history of colonoscopy, participation in routine health check-ups, and use of nonsteroidal anti-inflammatory drugs, with healthy lifestyle score ≤ 2 points as the reference in each PRS subgroup. CI, confidence intervals; CRC, colorectal cancer; OR, odds ratio; PRS, polygenic risk score; Ref., reference.

Table S8 Association of the healthy lifestyle score with colorectal cancer risk, stratified by age and gender

Variables	CRC cases, *n* (%)	Controls, *n* (%)	OR (95% CI)[1]	GRE (95% CI)	*P*-interaction[2]
**Age < 55 years**					0.083
Healthy lifestyle score					
0–2	158 (29.1)	78 (19.6)	Ref.	Ref.	
3	203 (37.4)	116 (29.1)	0.96 (0.65, 1.40)	−3.3 (−34.6, 27.9)	
4	128 (23.6)	127 (31.9)	0.51 (0.34, 0.77)	−55.1 (−95.2, −15.0)	
5	54 (9.9)	77 (19.3)	0.38 (0.23, 0.63)	−79.2 (−131.1, −27.2)	
Per 1-point increase			0.73 (0.64, 0.84)	−25.8 (−41.2, −10.3)	
PRS (per 10 percentile increase)			1.13 (1.07, 1.18)		
**Age ≥ 55 years**					
Healthy lifestyle score					
0–2	1,361 (31.9)	798 (22.5)	Ref.	Ref.	
3	1,512 (35.4)	1,221 (34.5)	0.77 (0.68, 0.88)	−21.4 (−32.3, −10.4)	
4	1,105 (25.9)	1,048 (29.6)	0.66 (0.57, 0.75)	−34.0 (−46.3, −21.7)	
5	293 (6.9)	476 (13.4)	0.39 (0.32, 0.47)	−77.0 (−96.2, −57.9)	
Per 1-point increase			0.79 (0.75, 0.83)	−19.3 (−24.1, −14.4)	
PRS (per 10 percentile increase)			1.13 (1.11, 1.15)		

Table S8 Continued

Variables	CRC cases, *n* (%)	Controls, *n* (%)	OR (95% CI)[1]	GRE (95% CI)	*P*-interaction[2]
**Female**					0.80
Healthy lifestyle score					
0–2	365 (19.4)	177 (11.7)	Ref.	Ref.	
3	649 (34.5)	457 (30.2)	0.71 (0.56, 0.90)	−28.0 (−48.1, −8.0)	
4	641 (34.1)	551 (36.5)	0.58 (0.46, 0.73)	−44.6 (−65.8, −23.3)	
5	227 (12.1)	326 (21.6)	0.36 (0.28, 0.48)	−83.6 (−111.9, −55.3)	
Per 1-point increase			0.75 (0.69, 0.81)	−23.5 (−31.6, −15.5)	
PRS (per 10 percentile increase)			1.13 (1.10, 1.16)		
**Male**					
Healthy lifestyle score					
0–2	1,154 (39.4)	699 (28.8)	Ref.	Ref.	
3	1,066 (36.4)	880 (36.2)	0.81 (0.70, 0.93)	−17.2 (−29.4, −5.1)	
4	592 (20.2)	624 (25.7)	0.66 (0.56, 0.78)	−34.0 (−48.6, −19.4)	
5	120 (4.1)	227 (9.3)	0.38 (0.29, 0.50)	−79.2 (−104.8, −53.5)	
Per 1-point increase			0.80 (0.75, 0.84)	−18.3 (−23.9, −12.6)	
PRS (per 10 percentile increase)			1.13 (1.11, 1.16)		

[1]Variables in the model included the healthy lifestyle score, age, gender, education, family history of CRC, history of colonoscopy, participation in routine health check-ups, use of nonsteroidal anti-inflammatory drugs, and PRS (per 10 percentiles, continuous) but without the stratification factors. [2]Interactions were tested by inclusion of a cross-product term of the healthy lifestyle score (categorical variable) and stratification factors in the models. CI, confidence intervals; CRC, colorectal cancer; GRE, genetic risk equivalent; OR, odds ratio; PRS, polygenic risk score; Ref., reference.

Table S9 Associations of the healthy lifestyle score with CRC risk, stratified by history of colonoscopy, use of NSAIDs, and family history of CRC

Healthy lifestyle score	CRC cases, *n* (%)	Controls, *n* (%)	OR (95% CI)[1]	GRE (95% CI)	*P*-interaction[2]
**History of colonoscopy: no**					0.22
Healthy lifestyle score					
0–2	1,155 (32.6)	352 (22.4)	Ref.	Ref.	
3	1,259 (35.5)	514 (32.8)	0.77 (0.65, 0.90)	−19.9 (−32.9, −7.0)	
4	878 (24.8)	483 (30.8)	0.58 (0.49, 0.69)	−41.6 (−56.7, −26.5)	
5	250 (7.1)	219 (14.0)	0.38 (0.30, 0.48)	−73.8 (−95.5, −52.2)	
Per 1-point increase			0.76 (0.71, 0.80)	−20.9 (−26.8, −15.1)	
PRS (per 10 percentile increase)			1.14 (1.12, 1.17)		
**History of colonoscopy: yes**					
Healthy lifestyle score					
0–2	364 (28.6)	524 (22.1)	Ref.	Ref.	
3	456 (35.8)	823 (34.7)	0.79 (0.66, 0.95)	−20.8 (−37.7, −3.9)	

Table S9 Continued

Healthy lifestyle score	CRC cases, *n* (%)	Controls, *n* (%)	OR (95% CI)[1]	GRE (95% CI)	*P*-interaction[2]
4	355 (27.9)	692 (29.2)	0.71 (0.58, 0.86)	−30.2 (−48.8, −11.6)	
5	97 (7.6)	334 (14.1)	0.39 (0.30, 0.52)	−83.1 (−113.9, −52.3)	
Per 1-point increase			0.81 (0.75, 0.86)	−18.6 (−26.0, −11.2)	
PRS (per 10 percentile increase)			1.12 (1.09, 1.15)		
**Use of NSAIDs: no**					0.55
Healthy lifestyle score					
0–2	1,039 (30.3)	503 (20.6)	Ref.	Ref.	
3	1,197 (34.9)	803 (32.9)	0.77 (0.66, 0.90)	−19.9 (−32.1, −7.8)	
4	927 (27.0)	741 (30.4)	0.64 (0.55, 0.75)	−34.1 (−47.4, −20.7)	
5	264 (7.7)	392 (16.1)	0.36 (0.29, 0.45)	−78.0 (−98.2, −57.8)	
Per 1-point increase			0.76 (0.72, 0.81)	−20.9 (−26.3, −15.6)	
PRS (per 10 percentile increase)			1.14 (1.12, 1.16)		
**Use of NSAIDs: yes**					
Healthy lifestyle score					
0–2	480 (34.6)	373 (24.8)	Ref.	Ref.	
3	518 (37.3)	534 (35.6)	0.80 (0.65, 0.97)	−19.7 (−38.0, −1.4)	
4	306 (22.1)	434 (28.9)	0.61 (0.49, 0.76)	−43.6 (−66.5, −20.7)	
5	83 (6.0)	161 (10.7)	0.46 (0.33, 0.63)	−68.5 (−102.5, −34.6)	
Per 1-point increase			0.80 (0.74, 0.87)	−19.7 (−28.3, −11.1)	
PRS (per 10 percentile increase)			1.12 (1.08, 1.15)		
**Family history of CRC: no**					0.0013
Healthy lifestyle score					
0–2	1,281(31.1)	801 (22.8)	Ref.	Ref.	
3	1,449 (35.3)	1,192 (33.9)	0.80 (0.70, 0.91)	−18.3 (−29.2, −7.3)	
4	1,075 (26.2)	1,028 (29.3)	0.69 (0.60, 0.79)	−30.4 (−42.6, −18.1)	
5	305 (7.4)	491 (14.0)	0.42 (0.35, 0.51)	−71.0 (−89.6, −52.4)	
Per 1-point increase			0.80 (0.76, 0.84)	−18.3 (−23.0, −13.5)	
PRS (per 10 percentile increase)			1.13 (1.11, 1.15)		
**Family history of CRC: yes**					
Healthy lifestyle score					
0–2	238 (33.8)	75 (17.4)	Ref.		
3	266 (37.8)	145 (33.8)	0.61 (0.42, 0.87)	−31.5 (−56.6, −6.4)	
4	158 (22.4)	147 (34.3)	0.32 (0.22, 0.47)	−72.6 (−105.1, −40.0)	
5	42 (6.0)	62 (14.5)	0.20 (0.11, 0.33)	−102.5 (−148.3, −56.7)	

Table S9 Continued

Healthy lifestyle score	CRC cases, *n* (%)	Controls, *n* (%)	OR (95% CI)[1]	GRE (95% CI)	*P*-interaction[2]
Per 1-point increase			0.59 (0.51, 0.68)	−33.6 (−47.0, −20.2)	
PRS (per 10 percentile increase)			1.17 (1.11, 1.23)		

[1]Variables in the model included healthy lifestyle scores, age, gender, education, family history of CRC, history of colonoscopy, participation in routine health check-ups, use of NSAIDs, and PRS (per 10 percentiles, continuous), but without the stratification factors. [2]Interactions were tested by inclusion of a cross-product term of the healthy lifestyle score (categorical variable) and stratification factors in the models. CI, confidence intervals; CRC, colorectal cancer; GRE, genetic risk equivalent; NSAID, nonsteroidal anti-inflammatory drug; OR, odds ratio; PRS, polygenic risk score; Ref., reference.
